# Another Theory on Phthisis

**Published:** 1848-07

**Authors:** 


*Another Theory on Phthisis.*—M. Wanner has communicated to the Académie de Médecine the result of certain investigations and experiments which he has made relative to the phthisical diathesis, by which he is led to believe that *calcareous* principles are the main cause of phthisis. M. Wanner had been informed that consumption was entirely unknown in the district of Sologne; he took up his residence in that part of the country for the space of fifteen months, and carefully investigated the subject. He found that a certain part of Sologne was really free from the slightest appearance of phthisis, scrofula, tabes mesenterica, or the calculous diathesis. The soil in this tract of land is, to a depth of about 240 feet, composed of silica and alumina, without the presence of calcareous matters; and the arable surface, which has very little depth, contains the same substances, with the addition of vegetable detritus. In some parts there is a certain amount of alluvial earth, but nowhere is there any lime to be detected. Those plants which thrive in calcareous soils are of course very stunted here; no vegetation flourishes but that which principally depends on silica—viz., rye, buck-wheat, some grasses, and a few potash and soda plants. From these facts, and the further study of the subject, the author concludes that tubercles owe their origin to the calcareous principles which are contained either in the solid food or in the different beverages which we make use of. The following is the author's classification of the causes of tuberculization:—

1. Aliments containing calcareous principles.

2. A certain state of the blood; whether the individual has been weakened by any extraneous cause, or by heredity.

3. Privation of exercise.

4. Damp dwellings and absence of light.

5. The introduction into the pulmonary organs of different kinds of dust.

Some therapeutic indications seemed to spring from these facts, and the author was induced to try the effect of large doses of carbonate of soda in the treatment of phthisis; but the attempt was unsuccessful. This medication seemed to him to be of benefit but to patients affected with chronic catarrh. The only practical advice which the author deduces from his long investigations is to recommend a residence in Sologne to those people who are labouring under a very recent tuberculous diathesis. We are anxious to see how the committee appointed by the Academy to report on this communication will take up the matter.—*Ibid.*

